# ‘A prospective case control study to evaluate shock index for identifying patients at risk of clinically important malaria in refugee settings’

**DOI:** 10.1177/00494755221134975

**Published:** 2023-01-18

**Authors:** Katy Kuhrt, Paul T Seed, Andrew H Shennan

**Affiliations:** 1 Department of Women and Children's Health, School of Life Course Sciences, Faculty of Life Sciences and Medicine, St Thomas’ Hospital, London, UK

**Keywords:** Malaria, community health formatted: English (United Kingdom)

## Abstract

Bidibidi Refugee Settlement's 223,000 refugees are vulnerable to malaria due to crowded conditions and limited healthcare access. Early identification and referral of suspected cases is key to reduce morbidity and mortality. We evaluated the shock index (heart rate/ systolic blood pressure) for detection of abnormal vital signs, calculated by the CRADLE Vital Signs Alert device, which can be used in routine patient blood pressure and heart rate assessment by non-medically trained Voluntary Health Team workers. The single most frequent diagnosis causing shock was malaria, and thus the device was useful to detect severe cases (as well as discovering other cases), after calculating appropriate shock indices.

## Introduction

Almost two-thirds of refugees live in malaria endemic regions.^1^ Uganda hosts 1.4 million refugees across 13 UN Refugee Agency (UNHCR) coordinated sites, including Bidibidi, the world's second largest refugee settlement, which spans 250km^2^ and hosts 223,000 refugees, mainly from South Sudan.^2^

Malaria is a leading cause of morbidity and mortality in Uganda, accounting for 30–50% of outpatient visits to health facilities and up to 20% of deaths.^3^ Refugees are particularly vulnerable due to proximity of the settlements to thick vegetation and stagnant water (favourable breeding grounds for mosquitoes), lack of availability of preventative measures including mosquito nets and insecticide and poor access to healthcare.^1^

Early detection and treatment are key to reducing the malaria burden.^4^ However, whilst the use of rapid diagnostic tests (RDTs) was implemented in many refugee settlements, according to UNHCR health information system (HIS) data, <45% of malaria cases are confirmed owing to limited access to tests.^1^ Furthermore there is a 90% shortfall in the health work force to deliver basic health care,^5^ leading to an increasing reliance on non-medically trained community volunteer Village Health Teamworkers, (VHTs), to perform community public health surveillance, including early identification of sick community members and prompt referral to health facilities for timely treatment. VHTs are currently poorly equipped for this role, in terms of training and equipment.^4^ The CRADLE Vital Signs Alert (VSA) is a cheap, portable, easy-to-use tool which measures blood pressure (BP), heart rate (HR) and calculates shock index (SI = HR/BP) and was originally designed for use in pregnant women, but has also been shown to be accurate in non-pregnant adults.^6–8^ It incorporates a built-in traffic light early-warning system to alert the user to abnormal vital signs and the need for timely referral. The traffic lights are triggered by standard thresholds of blood pressure, as well as by shock index, a proven marker of early deterioration secondary to trauma in combat settings,^9,10^ obstetric haemorrhage and sepsis.^11–13^

For example, a ‘red light’ and ‘up arrow’ indicates severe hypertension and should prompt intervention or referral; a ‘red light’ and a ‘down arrow’ secondary to shock also requires urgent attention; a ‘yellow light’ and either a ‘down arrow’ or ‘up arrow’ indicates a need for a non-urgent assessment or referral.

## Materials & methods

Ours was a prospective case control study to evaluate the CRADLE VSA for identification of patients at risk of clinically important malaria (defined by local staff) in the Bidibidi Refugee Settlement, Yumbe district, Northern Uganda. Ethical permissions and approval to carry out the work was granted by the Office of the Prime Minister and the UNHCR respectively.

All symptomatic patients aged 18 years or above presenting to a health worker (VHT in the community or a health worker based at a health facility), were eligible to participate. Individual patient consent was not required as CRADLE VSA measurements were taken as part of routine clinical activities. A total of 136 CRADLE VSAs were distributed to each of the 17 UNHCR health facilities (eight devices per health facility), and each VHT received their own device, between April and August 2018. This was supported by the CRADLE training package, delivered to all 100 health workers (health facility based and VHTs) including one off face-to-face training by the research team lasting approximately 5 h (presentation, short animated training films), and training materials: printed booklets and aide memoire cards attached to each device.

All participants had a CRADLE VSA reading performed as part of their routine assessment. If the results showed red or yellow the reading was repeated. If the yellow or red showed only once a third measurement was taken and the most consistent light was followed in terms of referral. Data were routinely collected (CRADLE VSA measurement including systolic blood pressure (SBP), diastolic blood pressure (DBP), HR and diagnosis (designated by the 6 attending health facility workers) from data record books at each health facility. Shock index was calculated for each patient. All cases were reviewed and grouped into ‘malaria’ and other disease categories. Malaria diagnoses were assigned after a positive rapid diagnostic test (according to health facility standard clinical practice). Additional analysis was performed on malaria cases defined as ‘severe’ by local clinical staff. A control group consisted of refugees undergoing asymptomatic screening using the CRADLE VSA in surrounding villages within the settlement.

Power calculation was based on the assumption of a 10% prevalence of malaria, and a sensitivity and specificity of 60% for shock index (positive likelihood ratio 1.5). We calculated that 660 subjects would be needed for 90% power. Mean shock index was calculated for malaria cases, other diseases and controls.

Predictive statistics, including positive and negative likelihood ratio (LR + /LR-), odds ratio and Receiver Operating Characteristic (ROC) area, were calculated to evaluate shock index for prediction of malaria and severe malaria. Positive and negative predictive values were not included as no reliable prevalence figures were available for severe malaria.

## Results

All of the 136 CRADLE VSA devices were successfully delivered to the health facilities and 451 VHTs received their own device. Data were collected on 3577 symptomatic cases (76% female) and 2163 asymptomatic controls (67% female) (Table 1). Mean shock index (SI) was 0.68 (95% confidence interval 0.67- 0.68) and 0.64 (95% confidence interval 0.63–0.65) in cases and controls respectively which was significantly different (0.039, 95% CI 0.03 to 0.0047).

**Table 1.** Demographic table to show characteristics of cases and control, malaria and other diseases groups.

Characteristic	Control, n (%)	Malaria, n (%)	Other disease categories, n (%)	All Groups, n (%)
n	2163	915	2662	5740
Mean Age	33	32	37	35
Female	1453 (67.2)	699 (76.4)	2024 (76)	4170 (72.7)
Male	710 (32.8)	216 (24)	638 (23.9)	1562 (27.3)
*CRADLE Vital Signs Alert Score*				
Green	1688 (78)	651 (71.2)	1879 (71)	4218 (73.5)
Yellow Up	312 (14.4)	100 (10.9)	379 (14)	791 (13.8)
Yellow Down	70 (3.2)	135 (14.8)	224 (8)	429 (7.5)
Red Up	93 (4.3)	29 (3.2)	180 (7)	302 (5.3)

Malaria accounted for 26% (915/3577) of cases, and had the highest mean SI compared to other diseases. Predictive statistics for all malaria cases and severe malaria only, based on a shock index threshold of 0.9 are shown in Table 2.

**Table 2.** Predictive statistics for all malaria cases vs severe malaria only.

Using a threshold of SI greater than or equal to 0.9 for a positive test	(95% Confidence Interval, p < 0.05)	
	All Malaria	Severe Malaria
n	929	38
Positive likelihood ratio	4.6 (3.5 to 6.10)	11.4 (7.1 to 18.3)
Negative likelihood ratio	0.88 (0.85 to 0.90)	0.65 (0.51 to 0.83)
Odds Ratio	5.3 (3.9 to 7.2)	17.44 (8.0 to 36.7)
ROC area	0.62 (0.60 to 0.65)	0.67 (0.56 to 0.77)

## Discussion

Shock index, calculated by the CRADLE VSA, could be used easily to identify patients likely to have malaria, and at the same time determine those most at risk of severe disease. The key to prevention of significant morbidity and mortality related to malaria is early detection. Although false negatives and positives are common with moderate ROC areas (Table 2), indicating that shock index does not represent a reliable diagnostic test, it has great clinical potential as a community based screening tool used by non-medically trained VHTs for early identification of those in need of attention. This could thus relieve the burden on medically trained health care workers, ensuring that scarce resources are targeted to the sickest patients. Such policy is well aligned with the Ugandan Ministry of Health growing emphasis on rapidly expanding and equipping health teams through VHTs to carry out community based surveillance including rapid detection and referral of suspected malaria cases and other life threatening conditions.

Previous work in India, Mozambique and South Africa has shown that unskilled community health workers perceive the CRADLE VSA as easy-to-use^14^; it meets specific WHO requirements for use in low income settings, being affordable (£19 British pound sterling), robust and portable with low power requirements.^15^ Further work in screening other refugee populations is needed.
